# Editorial: Current Topics in Opioid Research

**DOI:** 10.3389/fpsyt.2019.00586

**Published:** 2019-08-15

**Authors:** Lawrence Toll, Kelly M. Standifer, Dominique Massotte

**Affiliations:** ^1^Department of Biomedical Sciences, Charles E. Schmidt College of Medicine, Florida Atlantic University, Boca Raton, FL, United States; ^2^Department of Pharmaceutical Sciences, College of Pharmacy, University of Oklahoma Health Sciences Center, Oklahoma City, OK, United States; ^3^Centre de la Recherche Nationale Scientifique, Université de Strasbourg, Institut des Neurosciences Cellulaires et Intégratives (INCI) CNRS UPR3212, Strasbourg, France

**Keywords:** opioid, pain, drug abuse, review, pharmacology, imaging

Although natural opiates have been used for centuries, and semi-synthetic, and synthetic opiates have been used and abused for decades, these last few years have witnessed an incredible increase in opioid abuse and deaths due to overdose. With this opioid crisis has come an increase in research into the mechanisms of analgesia, abuse, and addiction, interaction with other drugs, anatomical and imaging studies, as well as ethical discussions of opioid use and abuse. This Research Topic, Current Topics in Opioid Research, presents 7 minireviews, 2 hypotheses/perspectives, and 16 original research articles, from 13 different countries, and has articles that span the field of opioid research, gives insight into ongoing topics, and provides a basis for further study and potential reduction in severity of the opioid crisis.

## Hypothesis and Theory/Perspective Articles

Drug Addiction: From Neuroscience to Ethics by Farisco et al. presents a novel hypothesis concerning drug addiction. The authors suggest that in addition to well-described neuronal/neurochemical factors contributing to addictive dynamics, the socioeconomic status also plays a causal role in drug addiction through epigenetic processes that require additional reward in the brain. This provides a strong base for a sociopolitical form of responsibility for preventing and managing the addiction crisis. For this reason, the authors consider addiction to be a social disorder in addition to a medical and mental disorder.

The Clinical Concept of Opioid Addiction Since 1877: Still Wanting After All These Years by Schütz et al. proposes a comprehensive theory of addiction that uses life and social sciences, dynamic and complex systems theory, and philosophical–phenomenological approaches to understand the full complexity of addiction while integrating neurobiological, psychological, and sociocultural aspects. According to this theory, addiction can be viewed as a habit, induced by a network of mental, behavioral, and social processes, which not only shape the addict’s perceptions and actions, but also cause one to self-maintain.

## Reward/Addiction

In Management of Opioid Addiction With Opioid Substitution Treatments: Beyond Methadone and Buprenorphine, Noble and Marie discuss drug therapies for addiction treatment. They discuss the benefits and detriments of the two mu opioid agonists, with a brief discussion of drug characteristics leading to these properties. They also discuss the use of the opioid antagonist naltrexone and potential leads toward next-generation medications, including the use of biased agonists, nociception/orphanin (NOP) receptor agonists, or potentially enkephalin degradation inhibitors.

In Enkephalin as a Pivotal Player in Neuroadaptations Related to Psychostimulant Addiction Mongi-Bragato et al. address changes in enkephalin levels in the mesocorticolimbic reward circuitry due to administration of psychostimulants. The authors discuss how these changes affect signaling of mu and delta opioid receptors and the importance of receptor activation with respect to cocaine- and amphetamine-induced behavioral sensitization, conditioned place preference (CPP), and self-administration.

Previously, Li et al. had demonstrated that activation of Trx-1, an important redox regulating protein, could protect mice from the rewarding effects of morphine. In Overexpression of Thioredoxin-1 Blocks Morphine-Induced Conditioned Place Preference Through Regulating the Interaction of γ-Aminobutyric Acid and Dopamine Systems, these authors demonstrate that morphine-induced CPP was blocked in Trx-1 overexpressing transgenic mice. Furthermore, Trx-1 expression was induced by morphine in the ventral tegmental area (VTA) and nucleus accumbens (NAc) in wild-type (WT) mice. The level of dopamine, expression of tyrosine hydroxylase (TH), and D1 dopamine receptor as well as levels of GABA and GABA_B-_ receptors were altered by chronic morphine. Therefore, Trx-1 may play a role in blocking CPP induced by morphine through regulating the expressions of D1, TH, and GABA_B_ receptors in the VTA and NAc.

Two papers described effects on drug abuse models subsequent to activation of NOP receptors, the fourth member of the opioid receptor family. In NOP receptor agonist Ro 64-6198 decreases escalation of cocaine self-administration in rats genetically selected for alcohol preference, Li et al. examine the effect of NOP receptor agonist Ro 64-6198 on alcohol self-administration in Marchigian Sardinian alcohol-preferring (msP) rats that have an upregulated NOP receptor system and in Wistar control rats. Ro 64-6198 was better able to attenuate cocaine self-administration in msP than in Wistar rats.

In The Nociceptin Receptor (NOP) Agonist AT-312 Blocks Acquisition of Morphine- and Cocaine-Induced Conditioned Place Preference in Mice, Zaveri et al. discuss the actions of AT-312, a selective NOP agonist, on morphine and cocaine CPP. AT-312 blocked acquisition of both morphine and cocaine as well as locomotor stimulation in WT but not NOP receptor knockout (KO) mice. These results demonstrate that NOP agonists may have a potential as pharmacotherapy for opioid and psychostimulant addiction or for treating polydrug addiction.

Three papers focused on the relationship between opiates and alcohol abuse. In Binge-Like Exposure to Ethanol Enhances Morphine’s Anti-nociception in B6 Mice, Chang et al. hypothesize that binge drinking potentiates onset and progression of opioid use disorder (OUD). To examine this, the authors examined and found an increase in inflammatory cytokines and mu receptor mRNA in the striatum after binge ethanol drinking. This corresponded with an increase in potency of morphine at 3 mg/kg in the hotplate test. Such effect might initiate the onset and progression of OUDs.

Granholm et al. described the effects of ethanol exposure on the level of opioid peptides in Episodic Ethanol Exposure in Adolescent Rats Causes Residual Alterations in Endogenous Opioid Peptides. To mimic binge drinking in adolescents, the authors administered ethanol to rats 3 days per week from weeks 4 to 9. Beta-endorphin, dynorphin B, and Met-enkephalin-Arg^6^Phe^7^ (MEAP) were then analyzed 2 h and 3 weeks after the final ethanol administration. Changes were observed for each peptide in selected brain regions. These alterations in opioid networks after adolescent ethanol exposure could explain, in part, the increased risk for high ethanol consumption later in life.

In Critical Role for Gi/o-Protein Activity in the Dorsal Striatum in the Reduction of Voluntary Alcohol Intake in C57Bl/6 Mice, Robins et al. explore the hypothesis that dorsal striatal Gi/o-protein activation is sufficient to reduce voluntary alcohol intake. The authors examined this hypothesis in two ways. In one set of experiments, they expressed the inhibitory, Gi/o-coupled, M4 DREADD in the dorsal striatum. In these animals, receptor activation with CNO reduced consumption of 10% ethanol in a two-bottle choice paradigm. In other experiments, delta opioid receptor activation with the Gi/o-biased agonist TAN-67 reduced alcohol consumption in WT and β-arrestin-2 KO animals, while activation with the β-arrestin-2-biased agonist SNC80 increased alcohol intake in WT but decreased intake in β-arrestin-2 KO animals. These results suggest that activation of Gi/o-coupled receptors in the striatum, with biased agonists, could be a mechanism for treating alcohol use disorder.

In CB1 Agonism Alters Addiction-Related Behaviors in Mice Lacking Mu or Delta Opioid Receptors, Roeckel et al. investigate the interaction between opioid and cannabinoid CB1 receptors with respect to pain, withdrawal, anxiety, and depression using the selective CB1 agonist ACEA, as well as mu and delta opioid receptor KO mice. The authors demonstrated that ACEA had no antinociceptive activity of its own in the warmwater tail withdrawal test. Naloxone was able to precipitate withdrawal from chronic ACEA in mice of all genotypes. Anxiety-like behavior was independent of genotype and ACEA treatment, but a pro-depressive effect of ACEA was absent in mu KO mice. These studies indicate an interaction between opioid and CB1 receptors in withdrawal and depression.

Butelman et al. examine opioid-dependent patients to determine if other drug use was a predictor of ultimate opioid dependence. In Non-medical Cannabis Self-Exposure as a Dimensional Predictor of Opioid Dependence Diagnosis: A Propensity Score Matched Analysis, the authors used an outpatient observational study to examine age of onset of heaviest use of cannabis, cocaine, and alcohol and how that correlated with the onset of opioid dependence. They concluded that the maximal self-exposure to cannabis and cocaine, but not to alcohol, was greater in volunteers with opioid dependence and that increasing self-exposure to cannabis and cocaine, but not alcohol, was a positive predictor of opioid dependence.

## Pain

Pain Therapy Guided by Purpose and Perspective in Light of the Opioid Epidemic, by Severino et al., discusses the history behind the current opioid epidemic then goes on to review potential methods for treatment of pain without off-target effects. To this end, the authors discuss the potential for ligand bias and bifunctional opioid agonists as potential methods of reducing side effects and also outline how the pharmacokinetic profile of opioids contribute to their potential for addiction and abuse.

In The Contribution of the Descending Pain Modulatory Pathway in Opioid Tolerance, Lueptow et al. review mechanisms that underlie opioid tolerance development, concentrating on the descending periaqueductal gray matter (PAG)–rostral ventromedial medulla (RVM)–spinal cord pain pathway. The authors describe how tolerance in the PAG is mediated by mu receptor uncoupling from downstream G-protein mediated signaling. Other experiments describe the relationship between tolerance development and glial activation; particularly, the role of TLR4 in that relationship is also discussed.

In Cutting-Edge Search for Safer Opioid Pain Relief: Retrospective Review of Salvinorin A and Its Analogs, Zjawiony et al. review the development of salvinorin A analogs from the perspective of a medicinal chemist. Salvinorin A, a natural product purified from *Salvia divinorum*, is a selective and high-affinity kappa agonist. *Salvia* is currently used as a recreational drug, since it alters consciousness but, like kappa drugs, is often dysphoric. Nevertheless, it has been used as a template for drug discovery leading to the production of many kappa and mu selective agonists. In particular, many analogs appear to have ligand bias, which might be useful in developing drugs with lower abuse potential.

In Advances in Achieving Opioid Analgesia Without Side Effects, Machelska and Celik review emerging opioid-based strategies to develop effective analgesics with reduced side effect profile. The novel concepts discussed include biased agonism, peripherally active compounds, heteromeric compounds, receptor splice variants, and use of endogenous opioids by inhibiting degradation or enhancing production. Compounds in clinical trials and undergoing preclinical studies are identified.

Zhang et al. examined the involvement of NOP receptors on a post-traumatic stress disorder (PTSD) model in male and female rats in Sex Differences in Nociceptin/Orphanin FQ Peptide Receptor-Mediated Pain and Anxiety Symptoms in a Preclinical Model of Post-traumatic Stress. Interestingly, male NOP receptor KO rats did not develop single prolonged stress-induced allodynia and thermal hypersensitivity, while female NOP receptor KO rats exhibited tactile allodynia and thermal hypersensitivity to the same extent as WT rats. These experiments demonstrate the distinct role that the NOP receptor system plays in males and females after exposure to sustained stress.

Stötzner et al., in Mu-Opioid Receptor Agonist Induces Kir3 Currents in Mouse Peripheral Sensory Neurons—Effects of Nerve Injury explore the activity of mu receptors in the dorsal root ganglia (DRG) to examine the potential of peripherally active mu agonists as analgesics with greatly reduced side effects. Receptor activity was determined by measuring activity of the G protein-coupled inwardly rectifying potassium channels, Kir3 in the DRG of naïve and chronic pain (chronic constriction injury CCI) mice. The authors determined that the mu agonist DAMGO could activate Kir3 currents and the percentage of mu-containing DRG neurons was equivalent in naïve and CCI mice.

## Pain/Novel Ligands

In *In Vitro* and *in Vivo* Pharmacological Activities of 14-O-Phenylpropyloxymorphone, a Potent Mixed Mu/Delta/Kappa-Opioid Receptor Agonist With Reduced Constipation in Mice, Lattanzi et al. discuss *in vitro* and *in vivo* properties of this novel compound (POMO). POMO is a 14-*O*-phenylpropyl-substituted analog of the mu opioid agonist 14-*O*-methyloxymorphone (14-OMO). POMO has subnanomolar affinity at mu, delta, and kappa receptors and is a full agonist at mu and delta. Importantly, it is a very potent analgesic, with an ED_50_ of 0.7 nmol/kg, 9,000 times more potent than morphine. Despite the extremely potent antinociceptive activity, it has reduced inhibition of gut transport, suggesting a greater therapeutic window.

Kumar et al. performed a structure–activity relationship study of oxymorphinone analogs in Synthesis, Biological Evaluation, and SAR Studies of 14β-phenylacetyl Substituted 17-cyclopropylmethyl-7,8-dihydronoroxymorphinones Derivatives: Ligands With Mixed NOP and Opioid Receptor Profile. Affinity and *in vitro* efficacy were determined at mu, delta, kappa, and NOP receptors. All compounds were partial agonists at each receptor, and structure activity relationship results were consistent with molecular modeling predictions that a binding site within the NOP receptor could be accessed by an appropriate 14β side chain. This resulted in compounds with much higher affinity at NOP receptors than the parent compound naltrexone.

## Mu Receptor

Heteromerization Modulates mu Opioid Receptor Functional Properties *in vivo* by Ugur et al. reviews the evidence for heteromers of the mu receptor and discusses how heteromers affect mu opioid receptor signaling, trafficking, and subsequent behavioral responses. The authors describe how selective targeting of heteromers to modulate mu opioid receptor activity has attracted significant interest as a method for developing novel innovative therapeutics.

In Microglia Express Mu Opioid Receptor: Insights From Transcriptomics and Fluorescent Reporter Mice, Maduna et al. examine microglia for the presence of the mu receptor. The reasoning behind these experiments was the observation that microglia appear to mediate certain adverse effects of opiates including analgesic tolerance and opioid-induced hyperalgesia. Using transcriptomic databases from both mice and humans, as well as imaging of newly created Cx3cr1l-eGFP-MOR-mCherry mice, the mu receptor was found in the vast majority of mouse and human microglia datasets. Furthermore, mu receptors could be found in roughly 40% of microglia in both brain and spinal cord. These results are consistent with functional studies showing the actions of mu receptor agonists on microglia.

In Oxycodone Self-Administration Induces Alterations in Expression of Integrin, Semaphorin and Ephrin Genes in the Mouse Striatum, Yuferov et al. examined the effect of chronic oxycodone treatment on molecules that affect axon guidance including integrin, semaphorin, and ephrin gene families. It was the author’s hypothesis that opioid-induced changes in axon-target connections and synaptogenesis may be implicated in the behaviors associated with opiate addiction. Chronic oxycodone caused either an increase or decrease in the majority of the 38 known genes in these gene families. The relationship between expression of these genes and specific behaviors is under investigation.

## Mu Receptor Imaging

In Deformation-based Morphometry MRI Reveals Brain Structural Modifications in Living Mu Opioid Receptor Knockout Mice, Nasseef et al. used a structural magnetic resonance imaging (MRI) approach to determine whether volumetric alterations also occur in mu opioid receptor KO mice. The authors measured deformation-based morphometry (DBM) for each voxel in subjects from mu KO and control groups. They found volumetric changes, both contractions and expansions in various brain regions, mainly in mu receptor-enriched regions and across reward/aversion centers. Some volumetric changes were in regions that showed functional connectivity changes identified in a previous resting-state functional MRI study, suggesting a possible function–structure relationship in mu KO-related brain alternations. These functional and structural MRI studies disclose whole-brain level mechanisms that likely drive mu-controlled behaviors.

Sasaki et al. demonstrated changes in tissue volume in the PAG using RMI voxel-based morphometry. In Larger Numbers of Glial and Neuronal Cells in the Periaqueductal Gray Matter of μ-Opioid Receptor Knockout Mice, these authors used immunohistochemistry to measure numbers of microglia, astrocytes, and neurons in four subregions of the PAG. They found larger numbers of each of these cell types in mu KO compared with WT mice, suggesting that these alterations might account for the hyperalgesic state in mu KO mice.

## Author Contributions

LT, KS, and DM all contributed to writing this Editorial of the Research Topic on Opioids that they edited in 2018.

## Funding

This work was supported by NIH grant DA023281 (LT), Richard T. Anderson Chair in Neuroscience Endowment and the Oklahoma Center for the Advancement of Science and Technology (OCAST HR17-041) (KMS) and CNRS (DM).

## Conflict of Interest Statement

The authors declare that the research was conducted in the absence of any commercial or financial relationships that could be construed as a potential conflict of interest.

